# A nationwide survey of intraoperative management for one-lung ventilation in Taiwan: time to accountable for diversity in protective lung ventilation

**DOI:** 10.1186/s12871-020-01157-w

**Published:** 2020-09-16

**Authors:** Chuan-Yi Kuo, Ying-Tung Liu, Tzu-Shan Chen, Chen-Fuh Lam, Ming-Cheng Wu

**Affiliations:** 1 Department of Anesthesiology, E-Da Hospital and E-Da Cancer Hospital, Kaohsiung, Taiwan; 2 Division of Respiratory Care, E-Da Hospital and E-Da Cancer Hospital, Kaohsiung, Taiwan; 3 Department of Medical Research, E-Da Hospital and E-Da Cancer Hospital, Kaohsiung, Taiwan; 4 School of Medicine, I-Shou University College of Medicine, Kaohsiung, Taiwan

**Keywords:** Airway management, Lung protective ventilation, One-lung ventilation, Postoperative pain management, Thoracic anesthesia

## Abstract

**Background:**

There is a major paradigm shift for intraoperative mechanical ventilator support by the introduction of lung protective ventilation strategies to reduce postoperative pulmonary complications and improve overall clinical outcomes in non-thoracic surgeries. However, there is currently a lack of standardized practice guideline for lung protection during thoracic surgeries that require one-lung ventilation (OLV). This study aimed to collect the expert opinions of the thoracic anesthesiologists in perioperative care for OLV surgery in Taiwan.

**Methods:**

This prospective cross-sectional study was undertaken in 16 tertiary hospitals in Taiwan from January to February 2019. A structured survey form was distributed across the participating hospitals and the thoracic anesthesiologists were invited to complete the form voluntarily. The survey form consisted of three parts, including the basic information of the institutional anesthesia care standards, ventilatory settings for a proposed patient receiving OLV surgery and expert opinions on OLV.

**Results:**

A total of 71 thoracic anesthesiologists responded to the survey. Double-lumen tubes are the most commonly used (93.8%) airway devices for OLV. The most commonly recommended ventilator setting during OLV is a tidal volume of 6–7 ml/kg PBW (67.6%) and a PEEP level of 4–6 cmH_2_O (73.5%). Dual controlled ventilator modes are used by 44.1% of the anesthesiologists. During OLV, high oxygen fraction (FiO_2_ > 0.8) is more commonly supplemented to achieve an oxygen saturation higher than 94%. The consensus of anesthesiologists on the indices for lung protection in thoracic surgery is considerably low. Large majority of the anesthesiologists (91.5%) highly recommend that an international clinical practice guideline on the protective lung ventilation strategy for thoracic anesthesia should be established.

**Conclusions:**

This study found that the thoracic anesthesiologists in Taiwan share certain common practices in ventilator support during OLV. However, they are concerned about the lack of fundamental clinical evidences to support the beneficial outcomes of the current lung protective strategies applicable to OLV. Large-scale trials are needed to form an evidence-based clinical practice guideline for thoracic anesthesia.

## Background

One-lung ventilation (OLV) is the foremost used technique of ventilation during thoracic procedures. Intraoperative lung separation can be managed by means of double-lumen endotracheal tube (DLT), bronchial blocker (BB), or nonintubated method [1, 2]. OLV is impeded by significant reduction in lung volume, decline in lung compliance at lateral decubital position, formation of intrapulmonary shunting and exposure of the dependent lung to ventilator-induced lung injury (VILI) [3]. In addition, patients receiving thoracic surgeries are more prone to developing acute lung injuries due to direct surgery-related trauma caused by instrumentation or manipulation of the lung tissues, hypoperfusion induced by hypoxic pulmonary vasoconstriction, and dysfunction of surfactant system [4]. The non-dependent lung is injured by surgical manipulation and atelectrauma. Re-expansion of the collapsed non-dependent lung at the end of surgery inevitably results in systemic inflammatory response in the local and contralateral lungs, which in turn leads to biotrauma [3, 5]. Therefore, a significantly high pulmonary complication of up to 14–28.4% was reported in patients that received OLV surgery [6].

In the recent two decades, there is a major paradigm shift for mechanical ventilator support during operation by the introduction of intraoperative lung protective ventilation strategies. Some of these changes include a low tidal volume (Vt), moderate levels of positive end-expiratory pressure (PEEP), optimal driving pressure (∆P) and the appropriate use of lung recruitment maneuver [7]. Intraoperative lung protective ventilation strategies have been shown to reduce post-operative pulmonary complications and improve overall clinical outcomes in intermediate and high-risk patients undergoing major abdominal surgery [7–9]. Currently, however, there is a lack of clinical evidence in regard to appropriate protective-lung strategies during OLV. The optimal levels of intraoperative use of oxygen fraction, the ventilatory settings for volume and pressure variables during OLV and re-expansion phases for lung recruitment are debating. Furthermore, diversities in clinical practice on airway management, advanced monitoring systems and pain control strategies for thoracic surgery are also observed.

Since the international clinical practice guidelines for intraoperative OLV are yet to be established, we conducted a nationwide survey among the thoracic anesthesiologists in Taiwan to determine the current status in practicing ventilatory support and anesthesia care during OLV surgery and analyze the levels of agreement in the intraoperative ventilatory settings among the thoracic anesthesiologists.

## Methods

The study was approved by the ethics committee and the institutional review board of E-Da hospital, Kaohsiung, Taiwan (Approval number EMRP-107-114). We conducted a physician-based, cross-sectional survey among 16 university hospitals or tertiary medical centers in Taiwan from 1 January 2019 to 28 February 2019. A structured survey form was distributed to the participated hospitals, and the thoracic anesthesiologists were invited to complete the survey voluntarily. The survey was developed by an expert panel that consisted of three anesthesiologists, a respiratory therapist, an intensivist and a biostatistician. The expert panel performed a systemic review of the current recommendations for perioperative ventilatory support, identified the common practice standards of perioperative ventilatory care in Taiwan, designed and validated the survey questionnaire, and structured the study design. To select a representative sample size for participation of this study and to optimize the loading of data collection, 16 hospitals (72.7%) were selected from the 22 tertiary referral medical centers according to the geographical regions of Taiwan. These participated hospitals contribute to about 58.4% of all thoracic surgery cases performed in Taiwan, while the rest of the cases (41.6%) are undertaken in the other 139 general hospitals across Taiwan.

The survey form consisted of three parts. The first part of the survey recorded basic information on the institute, such as annual caseload, institutional anesthesia care standards, and the numbers of thoracic anesthesiologists. A thoracic anesthesiologist was defined as a registered anesthesiologist who is committed to thoracic anesthesia service for at least two working days a week.

The second part of the survey investigated the preferred intraoperative ventilatory settings for a proposed female patient with a body mass index of 27 kg/m^2^ receiving video-assisted thoracoscopic lobectomy for right lung tumor. The thoracic anesthesiologists were asked to manage the ventilatory settings, fraction of inspiratory oxygen, ventilatory mode, and lung recruitment application during and after OLV.

The third part of the survey surveyed the thoracic anesthesiologists’ expert opinions on the need for clinical practice guidelines or recommendations on protective ventilation during OLV. The thoracic anesthesiologists were also asked to subjectively rank the importance of various ventilatory parameters that could be lung protective during OLV.

Since there was no group comparison in the study design, all findings are presented as descriptive data without comparative statistical analysis.

## Results

There was a total of 367 registered anesthesiologists in the 16 participated hospitals across the four cardinal regions of Taiwan, and 71 of these anesthesia specialists (19.3%) were on regular thoracic anesthesia service for at least two working days a week (which we defined as thoracic anesthesiologists). The response rate of this study was 95.8%, as there were three participants did not complete the second part of survey form. Table 1 shows the general information of the anesthesia care standard for thoracic surgery in each institute. Eight of these hospitals (50%) undertake more than 1000 thoracic surgeries each year (Table 1). Double-lumen endotracheal tube is the first-choice airway device (93.8%) for intraoperative lung separation, and most of the institutes employ intravenous patient-controlled analgesia (IVPCA) as the first-line pain control method after thoracic surgery (Table 1). Arterial catheterization is the standard intraoperative invasive hemodynamic monitoring system recommended for thoracic surgery, and 87.5% of institutes routinely apply bispectral index (BIS) for the monitoring of anesthetic depth (Table 1).

**Table 1:** The basic information of the anesthesia care standard for thoracic surgery

Cases of thoracic surgeries per year	
> 1000	50.0%
≤ 1000	50.0%
Lung isolation techniques for OLV	
Double-lumen endotracheal tube	93.8%
Bronchial blockers	6.3%
Laryngeal mask	0
Non-intubation	0
Postoperative analgesic management for OLV	
Intravenous patient-controlled analgesia	50.0%
Intercostal block	25.0%
Epidural analgesia	18.8%
Intravenous nonsteroidal antiinflammatory drug	6.3%
Paravertebral block	0
Perioperative monitoring systems during OLV	
Arterial catheter	100%
Bispectral index	87.5%
Central venous catheter	43.8%
Non-calibrated cardiac output monitor	12.5%
Pulmonary artery catheter	0

**Fig. 1:**
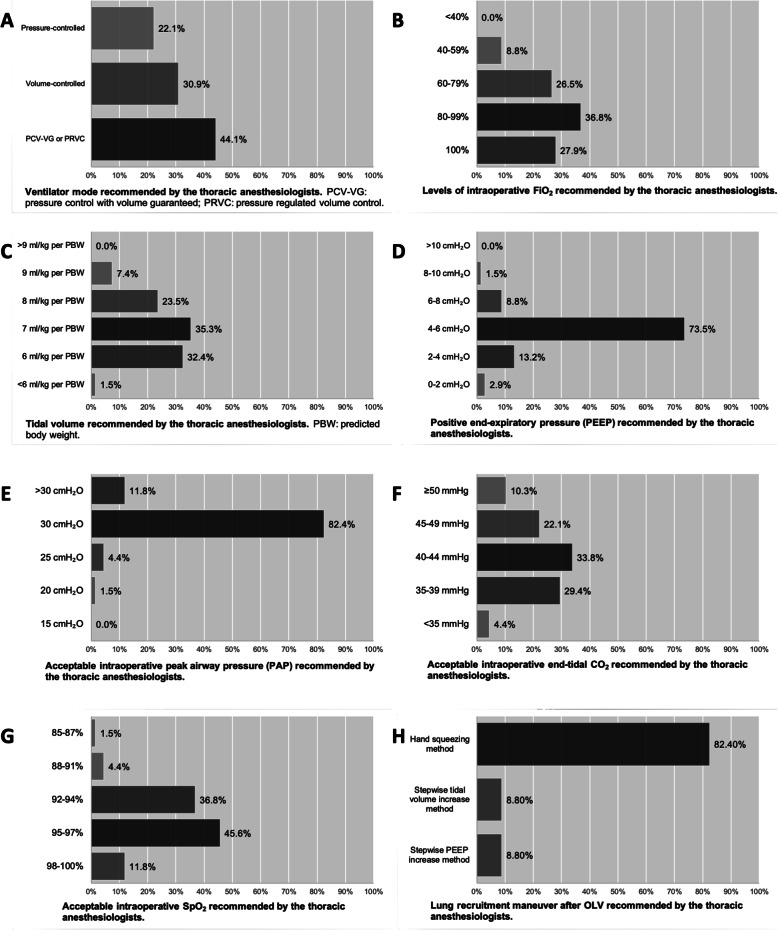
The intraoperative ventilatory settings recommended by the thoracic anesthesiologists

Part II of the form surveyed the intraoperative ventilatory settings managed by the thoracic anesthesiologists regarding a female patient with a predicted body weight (PBW) of 51 kg who was proposed to receive OLV for right middle and lower lung lobectomy (Supplementary form 1). 44.1% (30/68) of the anesthesiologists applied the dual controlled ventilator modes (i.e. pressure control with volume guaranteed (PCV-VG) or pressure regulated volume control (PRVC) mode) for OLV support; while 30.9% (21/68) and 22.1% (15/68) of the responders used the conventional volume-controlled and pressure-controlled modes, respectively (Fig. 1). High inspiratory fractions of oxygen (FiO_2_ > 80%) were more commonly administered during OLV (64.7%) (Fig. 1). Most of the anesthesiologists ventilated the patient with a Vt of 6–8 ml/kg/PBW (91.1%, 62/68) and a PEEP of 2–6 cmH_2_O (86.8%, 59/68) (Fig. 1). A peak airway pressure (PAP) less than 30 cmH_2_O was considered to be clinically acceptable in the dependent lung during OLV (Fig. 1). Only few anesthesiologists permitted levels of expiratory CO_2_ (EtCO_2_) greater than 50 mmHg (10.3%, 7/68) and levels of peripheral oxygen saturation (SpO_2_) lower than 94% (42.6%, 29/68) during operation (Fig. 1). 42.6% (29/68) of the responders would consider reducing FiO_2_ when a SpO_2_ ≥ 98% was measured (Fig. 1). At the end of OLV, hand squeezing method was the most commonly used maneuver to recruit the non-dependent lung (82.4%, 56/68) (Fig. 1).

**Fig. 2:**
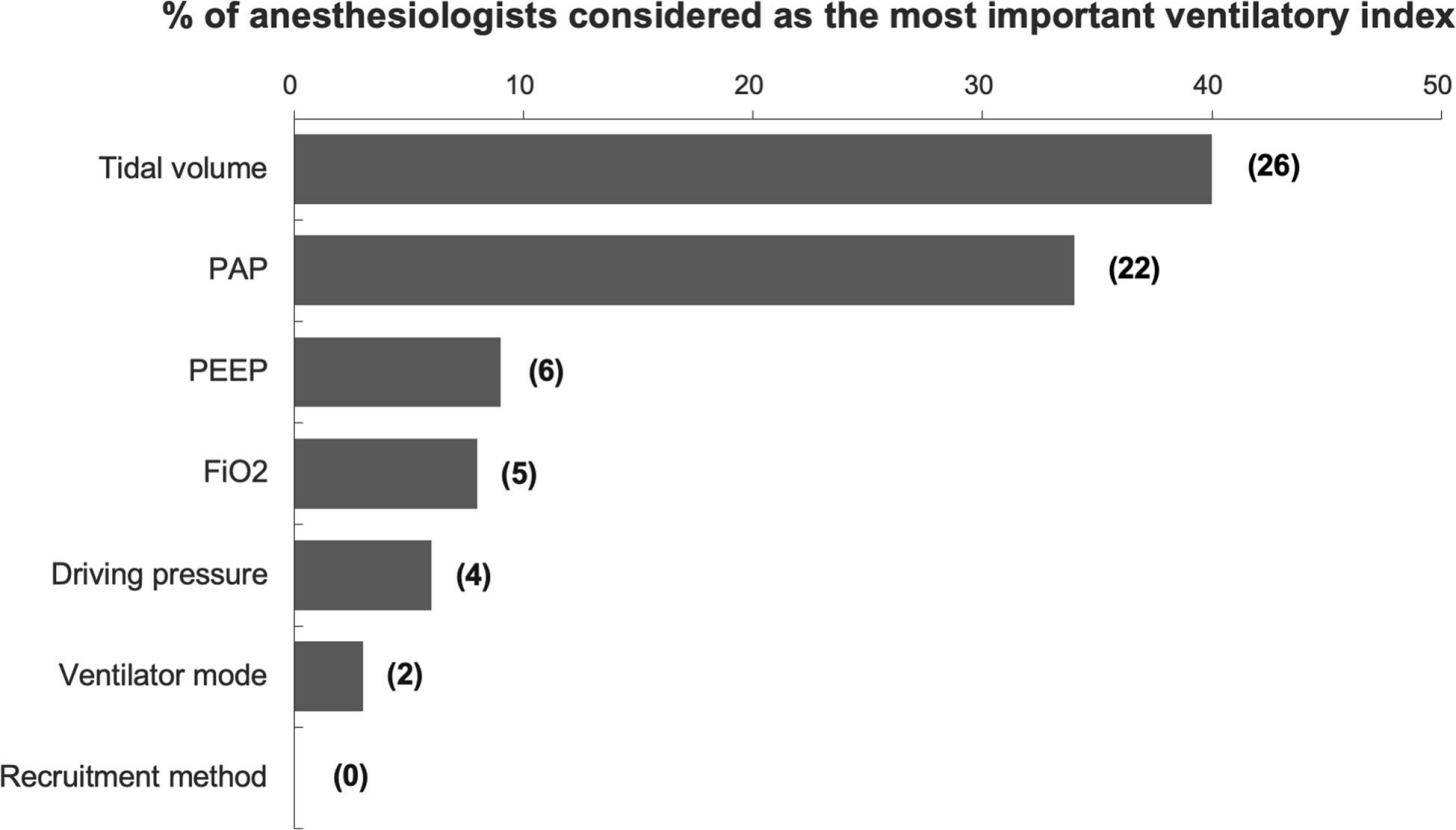
The most important ventilatory parameters that considered by the thoracic anesthesiologists as lung protective during one-lung ventilation. Numbers in the brackets indicate numbers of the thoracic anesthesiologists. FiO_2_: Inspiratory fractions of oxygen; PAP: Peak airway pressure; PEEP: Positive end-expiratory pressure

Part III of the survey form collected the expert opinions of the thoracic anesthesiologists in the protective lung ventilation strategy during OLV. Optimal levels of Vt and PAP were suggested as the two most important ventilatory parameters for lung protection during OLV (Fig. 2). However, there were as high as 60.0 and 66.2% of the thoracic anesthesiologists did not consider Vt and PAP as the most important ventilator indices for lung protection during OLV, respectively (Fig. 2). Furthermore, the proportions of these experts who considered other parameters (i.e. PEEP, FiO2, ∆P, ventilator mode and recruitment maneuver) as the most important indexes to guide the ventilatory strategy for lung protection during thoracic surgery were extremely low (Fig. 2). Most importantly, 91.5% (65/71) of the thoracic anesthesiologists highly recommended that an international clinical practice guideline on the protective lung ventilation strategy for thoracic anesthesia should be established.

## Discussion

This survey indicates that most centers in Taiwan employ DLT for OLV. Arterial catheter and BIS are the common perioperative monitoring systems used in Taiwanese centers during thoracic surgery. 50% of these centers consider IVPCA for postoperative pain control. During OLV, most thoracic anesthesiologists recommended high oxygen fraction supplement (FiO_2_ > 80%) and ventilated the patients with a tidal volume of 6–8 ml/kg/PBW and a PEEP of 2–6 cmH_2_O using the dual-controlled mode. A PAP less than 30 cmH_2_O is considered the threshold to avoid barotrauma. Most thoracic anesthesiologists try to maintain relatively normal levels of CO_2_ and SpO_2_ during OLV. Manual hand squeezing method is more often used for lung recruitment at the end of operation. The Taiwanese thoracic anesthesiologists urge for an international practice guideline for protective lung ventilation during OLV.

Two other nationwide surveys were reported by the Italian and Taiwanese groups [1, 10]. These two retrospective studies found that more than 90% of the Italian and Taiwanese centers used a DLT as their first choice for intraoperative OLV (90–96%) [1, 10]. Consistent with these previous reports, our study also found that only 6.2% of the Taiwanese thoracic anesthesiologists would prefer to use a bronchial blocker for selective lung ventilation during thoracic surgery. A total of 39% of the Italian centers recommended epidural analgesia for postoperative pain management [1]. Our study found that 43.8% of the participated centers performed loco-regional blocks for postoperative pain control. According to the American Pain Society clinical practice guidelines, thoracic epidural analgesia is considered as the most effective route for thoracic pain control and should be routinely considered for management of surgical pain after thoracotomy [11]. In the recent two decades, minimally invasive video-assisted thoracoscopic surgery (VATS), which is associated with minimal tissue injury, have been widely adapted by the thoracic surgeons. Therefore, less invasive loco-regional techniques are the more favorable approaches than epidural analgesia for VATS [12]. Since majority of the thoracic surgeries are currently underwent using the minimally invasive techniques in Taiwan [13], the fact that more than half of these centers used parenteral analgesic techniques instead of epidural analgesia as the first-line analgesia method is therefore reasonable. Nevertheless, loco-regional block techniques, such as paravertebral block, intercostal block and serratus anterior plane block, for perioperative pain control after VATS or other minimally invasive thoracic procedures should be vigorously promoted in the Taiwan medical institutes in order to enhance more effective postoperative pain relief, shift toward opioid-free analgesia, and prevent the development of chronic pain syndromes [14]. Furthermore, direct comparisons of the anesthesia management for thoracic surgery between these studies might be inappropriate, as the standards of anesthesia care have changed after the introduction of enhanced recovery after surgery (ERAS) protocols [15] and other clinical pathways [16].

The main objective of this survey was to determine the strategies of ventilatory support during and after OLV. Compared with the Italian study reported 6 years ago, more Taiwanese thoracic anesthesiologists ventilated the patients using the dual-controlled ventilatory modes (PRVC or PCV-VG mode) during OLV, which might be a reflection of increased availability of these novel ventilatory modes in clinical anesthesia. These dual-controlled modes deliver the preset tidal volumes with lowest optimal airway pressure, which may theoretically reduce the risk of barotrauma [17]. Although several clinical studies have suggested that dual-controlled modes enhanced oxygenation parameters with improved respiratory mechanics during OLV in general population and elderly [18–20], large-scale clinical trials are needed to confirm the overall pulmonary protective outcomes of the dual-controlled ventilatory modes during OLV and at the lung recruitment phase.

Low tidal volume (6–8 ml/kg PBW) is one of the hallmark parameters for intraoperative lung protective ventilation during non-thoracic surgery [9]. However, the application of an “optimally low” tidal volume during OLV is not standardized. Our survey and other retrospective database analysis suggest that there are a considerably large proportion of patients continue to receive the similar range of tidal volume (6–8 ml/kg PBW) during two-lung and one-lung ventilation [21]. However, the level of tidal volume has been shown to be inversely related to the incidence of respiratory complications and major postoperative morbidity [21]. Furthermore, the Italian and Japanese anesthesiologists recommend a lower tidal volume (4–6 ml/kg PBW) for OLV [1, 22]. Nevertheless, opinions from the expert anesthesiologists highlight that protective ventilation in thoracic anesthesia is not simply synonymous of a low tidal volume, but also involves the appropriate application of PEEP, alveolar recruitment and other ventilatory settings during OLV [23, 24]. Most recently, a double-blind, randomized controlled trial conducted at the Samsung Medical Center (Seoul, Korea) demonstrated that driving pressure-guided ventilation (median ∆P of 9 cmH_2_O) during OLV significantly reduced the incidence of postoperative pulmonary complications compared with the conventional protective ventilation (tidal volume 6 ml/kg PBW, PEEP 5 cmH_2_O and recruitment) in thoracic surgery [25]. PEEP is another important element in practicing intraoperative lung protective ventilation. This survey found that most of the thoracic anesthesiologists in Taiwan apply a PEEP level of 4–6 cmH_2_O during OLV, which is comparable with mean levels (4.2 ± 1.6 cmH_2_O) reported in a large retrospective analysis of the US database [21]. The authors concluded that low tidal volume failed to reduce postoperative pulmonary complications without application of adequate PEEP [21]. A previous study also indicated that individualized PEEP determined by a PEEP decrement trial significantly increased oxygenation and lung mechanics than the standardized PEEP (5 cmH_2_O) [26]. However, the appropriate PEEP levels for OLV are yet to be determined by the ongoing clinical trials (Table 2). Our study also found that most Taiwanese thoracic anesthesiologists currently use the bag squeezing maneuver to recruit of the collapsed non-dependent lung. Although the stepwise recruitment methods have been shown to reduce the incidence of postoperative pulmonary complications in comparison to bag squeezing maneuver in abdominal surgery [27], the evidence for re-expansion methods for the non-dependent lung after OLV requires further investigation. In fact, the ongoing Prothor and iPROVE-OLV trials are analyzing the lung protective effects of high PEEP, recruitment maneuver, and postoperative high-flow nasal cannulas for thoracic surgeries requiring OLV (Table 2) [28].

**Table 2:** Ongoing ClinicalTrial.gov registered trials regarding OLV during thoracic surgery

Title of trial	Location of trial	Interventions and outcome measures	ClinicalTrial.gov registration #
Effect of Lung Protective One-lung Ventilation with Fix and Variable PEEP on Oxygenation and Outcome	Hungary, single center	Interventions Under tidal volume 6 mL/kg/PBW, compare fix 5 cmH_2_O PEEP and variable PEEP with recruitment maneuvers. Outcome Measures Primary: intraoperative oxygenation Secondary: postoperative complications and survival	NCT03968120
Optimal Level of PEEP in Protective One-lung Ventilation	Korea, single center	Interventions Under tidal volume 5 mL/kg/PBW, compare 3, 6, and 9 cmH_2_O PEEP and variable PEEP. Outcome Measures Primary: modified lung ultrasound score Secondary: intraoperative desaturation, PaO_2_/FiO_2_, plasma inflammatory cytokines, postoperative desaturation and pulmonary complication	NCT03856918
Electrical Impedance Tomography in One-Lung Ventilation	Chile, single center	Interventions Compare three tidal volumes (4, 6 and 8 mL/kg/PBW) and two PEEP’s (6 cmH_2_0 and best PEEP obtained after a recruitment maneuver and decremental titration). Outcome Measures Ventilation/perfusion ratio, pulmonary mechanics, arterial gas measurement	NCT03728010
Individualized vs Low PEEP in One Lung Ventilation	US, single center	Interventions Compare individualized PEEP (max lung compliance) and low PEEP (5 cmH_2_O). Outcome Measures Primary: cerebral oximetry Secondary: arterial and venous blood oxygen tension, venous blood oxygen saturation, cardiac output, phenylephrine dose	NCT03569774
Individualized Perioperative Open-Lung Ventilatory Strategy During One-Lung Ventilation (iPROVE-OLV)	International, multicenter	Interventions Under tidal volume 5–6 mL/kg/PBW, compare alveolar recruitment maneuver plus PEEP titration trial and lung protective ventilation (PEEP 5 cmH_2_O). Outcome Measures Primary: postoperative pulmonary complications Secondary: postoperative complications, length of hospital stay	NCT03182062
Protective Ventilation with High Versus Low PEEP During One-lung Ventilation for Thoracic Surgery (PROTHOR)	International, multicenter	Interventions Under tidal volume 5 mL/kg/PBW, compare higher PEEP (PEEP 10 cmH_2_O + lung recruitment) and lower PEEP (PEEP 5 cmH_2_O only). Outcome Measures Primary: postoperative pulmonary complications	NCT02963025

*PaO*_*2*_*/FiO*_*2*_ Partial pressure of arterial oxygen/fraction of inspired oxygen ratio, *PBW* Predicted body weight, *PEEP* Positive end-expiratory pressure, *OLV* One-lung ventilation

Collapse of non-dependent lung and atelectasis of dependent lung during OLV increases intrapulmonary shunt and leads to the development of intraoperative hypoxemia [29]. Therefore, higher oxygen fractions are more commonly supplemented during lung separation procedures than the non-thoracic surgeries [22, 30]. However, oxygen therapy in clinical anesthesia is considered as a two-edged sword and excessive oxygen supplement should be avoided to prevent the potential oxygen toxicity [31], as potentially preventable hyperoxemia is considered as a SpO_2_ greater than 98%, despite a FiO_2_ of more than 0.21 [30]. An observational study found that higher FiO_2_ during OLV was associated with significantly higher incidence of postoperative pulmonary complications (OR 1.30; 95% CI 1.04–1.65) [22]. High quality-controlled studies are thus essential to compare the clinical outcomes of low versus high fractions of oxygen used for OLV.

Current clinical practice guidelines recommend that a tidal volume of 6–8 ml/kg predicted body weight and an optimal PEEP of 5 cmH_2_O are the most important ventilatory indices to guide intraoperative lung protection during mechanical ventilation in general population and obese patients [32]. However, consensus on the individual ventilatory parameters that are considered as lung protective during OLV is still lacking. Our results found that the Taiwanese thoracic anesthesiologists concern that there is currently no common consensus in the intraoperative lung protective ventilation during thoracic surgery, particularly at the OLV phase. These anesthesiologists have diverse agreement to recommend the most important ventilator-derived parameters (i.e. tidal volume, PAP, PEEP and ∆P) for the guidance of lung protection during OLV (degrees of agreement: Vt > PAP > PEEP >FiO2 > ∆P > mode > recruitment; Fig. 2). In fact, a number of prospective randomized controlled trials are currently undertaking, including several international multicenter studies, to determine the strategy for lung protective ventilation during thoracic surgery (Table 2).

There are a number of limitations with this study. First, the case scenario of lung tumor proposed in section 2 of the questionnaire specified that the lung resection was performed with VATS. Therefore, the data collected in the study may not be applicable to anesthesia and ventilatory care for patients receiving open thoracotomies. Secondly, this study analyzed the opinions of anesthesiologists in perioperative care for general patients. Patients with other underlying disease, such as chronic obstructive pulmonary disease, may need individualized ventilatory support strategy for thoracic surgery. Thirdly, all responses were based on the expert opinion or clinical experience of the participating thoracic anesthesiologists and could be subject to respondent bias. The optimal ventilatory settings or indices during OLV (e.g. Vt, PAP, PEEP and FiO_2_) suggested by the anesthesiologists could be arbitrary or not evidence based. Fourthly, this study primarily aimed to analyze the expert opinions of thoracic anesthesiologists on the anesthesia care and ventilatory support during thoracic surgery requiring OLV. Other unexpected perioperative adverse events such as surgical-related injury, severe bleeding, unstable hemodynamics, hypothermia, delirium or drug-responses [33] that could influence the general outcomes of thoracic surgeries were not taken into consideration in this report. Lastly, these results might not be completely representative of the expert opinions of all the Taiwanese thoracic anesthesiologists, as some medical centers and other regional hospitals were not included in our study.

## Conclusions

The thoracic anesthesiologists in Taiwan share certain general consensuses in regard to the practice in the ventilatory care during thoracic anesthesia. However, the clinical evidence in supporting the beneficial outcomes of the current lung protective strategies used during OLV is apparently insufficient. Several large-scale clinical trials are currently undertaking in thoracic surgery to evaluate the pulmonary protective ventilatory strategy during OLV and lung recruitment. There is an essential need to make a call for generating evidence-based practice guideline regarding intraoperative lung protective ventilation for thoracic anesthesia.

## Supplementary information

**Additional file 1.** Survey form.

## Data Availability

The datasets used and/or analyzed during the current study are available from the corresponding author on reasonable request.

## Abbreviations

∆P: Driving pressure
BB: Bronchial blocker
BIS: Bispectral index
CI: Confidence interval
DLT: Double-lumen endotracheal tube
EtCO_2_: Expiratory carbon dioxide
FiO_2_: Inspiratory fractions of oxygen
IVPCA: Intravenous patient-controlled analgesia
OLV: One-lung ventilation
OR: Odds ratio
PAP: Peak airway pressure
PBW: Predicted body weight
PCV-VG: Pressure control with volume guaranteed
PEEP: Positive end-expiratory pressure
PRVC: Pressure regulated volume control
SpO_2_: Peripheral oxygen saturation
VILI: Ventilator-induced lung injury
Vt: Tidal volume
