# Supplementary material for: A nationwide survey of intraoperative management for one-lung ventilation in Taiwan: time to accountable for diversity in protective lung ventilation
Source: BMC Anesthesiol. 2020 Sep 16;20:236. doi: 10.1186/s12871-020-01157-w (PMC7493315; doi:10.1186/s12871-020-01157-w)
Supplement: Supplementary file 1 — Additional file 1. Survey form. [file 12871_2020_1157_MOESM1_ESM.docx]

Survey form

Part I: Basic information of the institute

1. The approximate numbers of thoracic surgeries performed in a year.
2. The first choice for lung isolation technique during one-lung ventilation:
3. Double-lumen endotracheal tube
4. Bronchial blockers
5. Laryngeal mask
6. Non-intubation
7. The first choice for analgesic management after thoracic surgery:
8. Intravenous patient-controlled analgesia
9. Epidural analgesia
10. Paravertebral block
11. Intercostal block
12. Other
13. The routine perioperative monitoring systems used for thoracic surgery (multiple-choice)
14. Arterial catheter
15. Central venous catheter
16. Pulmonary artery catheter
17. Non-calibrated cardiac output monitor
18. Bispectral index
19. Other

Part II: Ventilatory settings during OLV

Case scenario: A 48-year-old female (158cm, 68kg; BMI 27) is scheduled for right lower lobe (RLL) and right middle lobe (RML) lung lobectomy due to primary tumor. The patient denies any other systemic diseases. The peripheral oxygen saturation (SpO_2_) under room air is 98%. One-lung ventilation (OLV) is required during operation.

1. The ventilator mode you would choose during OLV:
2. Pressure-controlled
3. Volume-controlled
4. Pressure control with volume guaranteed (PCV-VG) or pressure regulated volume control (PRVC)
5. Other
6. The fraction of inspiratory oxygen (FiO2) supplement during OLV:
7. 100%
8. 80-99%
9. 60-79%
10. 40-59%
11. <40%
12. The tidal volume set for OLV?
13. <6 ml/kg per PBW
14. 6-7 ml/kg per PBW
15. 7-8 ml/kg per PBW
16. 8-9 ml/kg per PBW
17. >9 ml/kg per PBW
18. The positive end-expiratory pressure (PEEP) set for OLV:
19. 0-2 cmH2O
20. 2-4 cmH2O
21. 4-6 cmH2O
22. 6-8 cmH2O
23. 8-10 cmH2O
24. >10 cmH2O
25. The upper limit of peak airway pressure during OLV:
26. 15 cmH2O
27. 20 cmH2O
28. 25 cmH2O
29. 30 cmH2O
30. >30 cmH2O
31. The ranges of end-tidal CO_2_ acceptable during OLV:
32. <35 mmHg
33. 35-39 mmHg
34. 40-44 mmHg
35. 45-49 mmHg
36. ≥50 mmHg
37. The range of SpO_2_ acceptable during OLV?
38. 98-100%
39. 95-97%
40. 92-94%
41. 88-91%
42. <87%
43. Will FiO_2_ be reduced if SpO2 >98% during OLV?
44. No
45. Yes
46. The maneuvers used for lung recruitment after OLV:
47. Hand squeezing method
48. Stepwise tidal volume increase method
49. Stepwise PEEP increase method

Part III: Expert opinions on OLV

1. Do you think that a clinical practice guideline or consensus on protective lung ventilation during thoracic anesthesia is necessary?
2. No
3. Yes
4. Please score the following ventilator settings by the importance of these parameters for lung protection during OLV, according to your experience or opinion. “1” indicates the most importance, and “5” indicates the least importance.
5. Tidal volume
6. PEEP
7. Driving pressure
8. Peak airway pressure
9. FiO2
10. Ventilator mode
11. Lung Recruitment method
12. Other
